# Impact of extractive industries on malaria prevalence in the Democratic Republic of the Congo: a population-based cross-sectional study

**DOI:** 10.1038/s41598-022-05777-9

**Published:** 2022-02-02

**Authors:** Cedar L. Mitchell, Mark M. Janko, Melchior K. Mwandagalirwa, Antoinette K. Tshefu, Jessie K. Edwards, Brian W. Pence, Jonathan J. Juliano, Michael Emch

**Affiliations:** 1 Department of Epidemiology, Gillings School of Global Public Health, University of North Carolina, 135 Dauer Dr., Chapel Hill, NC 27599 USA; 2 Institute for Health Metrics and Evaluation, University of Washington, Seattle, WA USA; 3 Kinshasa School of Public Health, Hôpital General Provincial de Reference de Kinshasa, Kinshasa, Democratic Republic of Congo; 4 Division of Infectious Diseases, University of North Carolina School of Medicine, Chapel Hill, NC USA; 5 Department of Geography, University of North Carolina, Chapel Hill, NC USA

**Keywords:** Ecological epidemiology, Malaria

## Abstract

Extraction of natural resources through mining and logging activities provides revenue and employment across sub-Saharan Africa, a region with the highest burden of malaria globally. The extent to which mining and logging influence malaria transmission in Africa remains poorly understood. Here, we evaluate associations between mining, logging, and malaria in the high transmission setting of the Democratic Republic of the Congo using population-representative malaria survey results and geographic data for environmental features and mining and logging concessions. We find elevated malaria prevalence among individuals in rural areas exposed to mining; however, we also detect significant spatial confounding among locations. Upon correction, effect estimates for mining and logging shifted toward the null and we did not find sufficient evidence to detect an association with malaria. Our findings reveal a complex interplay between mining, logging, space, and malaria prevalence. While mining concessions alone may not drive the high prevalence, unobserved features of mining-exposed areas, such as human migration, changing vector populations, or parasite genetics, may instead be responsible.

## Introduction

Malaria remains a major cause of illness and mortality across sub-Saharan Africa with an estimated 215 million cases reported in 2019 resulting in over 384,000 deaths^1^. The high burden of malaria in Africa has been attributed to a complex network of proximal causes, but the region’s environmental suitability for high transmission of malaria is an undeniable root cause^2,3^. Disturbances to the natural environment through urbanization, deforestation, and agriculture have been shown to significantly alter malaria transmission paradigms, resulting in varied levels of the disease^3–6^. Extraction of natural resources through mining or logging also markedly changes natural environments and ecologies, but the effects of these larger scale government allocated operations, referred to as concessions, on malaria remain poorly characterized in sub-Saharan Africa.

Mining and logging industries provide critical streams of revenue and employment opportunities across much of Africa. Exportation of minerals alone accounts for an estimated 70% of the continent’s total exports^7^ and in Central Africa, nearly 50 million hectares of the Congo Basin are permitted for industrial logging concessions^8^. Extraction of minerals and timber disrupts the environment in ways that can intensify malaria transmission^6,9,10^. However, the economic gains from mining and logging activities often contribute to improved housing quality, strengthened infrastructure and access to healthcare, and higher incomes^8,11^—all of which help reduce the burden of malaria^4,12^. Additionally, many large mining corporations sponsor malaria control interventions to improve the health of their workforce^11,13–15^. Thus, while mining and logging activities undoubtedly shift local environmental and ecological dynamics, there is also increased potential that malaria transmission may be counterbalanced by the positive impact of these concessions on rural economies and employment stability.

The knowledge base regarding the effects of extractive industries on malaria largely stems from studies of gold mining and deforestation in South America^10,16–19^. In Venezuela and Suriname, a large percentage of malaria cases have been attributed to miners^20^, whose mobility and high risk exposure patterns challenge malaria detection and prevention efforts^16,20^. Of the limited malaria studies conducted in African mining regions^13,15,21,22^, only one examined associations between proximal residence to an active mining concession and malaria prevalence^22^. Knoblauch and colleagues identified a protective effect of close residence to a Zambian copper mine among children; however, their focus on a singular mine limits generalizability of their findings. Studies of deforestation in the Amazon basin have repeatedly linked forest loss to increased malaria^10,19^; however, associations between forest loss and malaria in Africa have been mixed. In the East African Highlands, decades of forest loss have been associated with increased malaria incidence^6,23^. A recent multi-national analysis of malaria and remotely sensed forest loss in the Congo Basin found a null effect of deforestation on malaria prevalence in children; however, their use of aggregate survey data may have precluded detection of associations at local scales^24^. Others have postulated that effects of forest loss on malaria may change over time as vector populations respond to the altered environment^25^.

Here, we conduct a population-based study to examine associations between exposure to mining and logging concessions and malaria prevalence in the Democratic Republic of the Congo (DRC). The DRC has the second highest prevalence of malaria in the world^1^, exhibits a booming mining sector^7,9^, and is home to the largest contiguous area of the Congo Rainforest^8^. The presence of mining and logging concessions and high occurrence of malaria in the DRC allows for an ideal setting in which to evaluate the effect of these extractive industries on malaria.

## Methods

### Study design

The primary data source for this study is the cross-sectional 2013–2014 Demographic and Health Surveys for the DRC which is joined with remote-sensed environmental measures and land use data for mining and logging concessions extracted to DHS survey cluster locations. The DHS was administered using a multi-stage cluster survey design to represent the population of the DRC^26^. Briefly, survey clusters were selected to be representative of all 26 DRC provinces. Within clusters, households were randomly selected proportional to the population size, and within each household, adults ages 15–59 years were consented, interviewed, and asked to provide a dried blood spot (DBS) sample. Only adults who provided a DBS and consented for biospecimen use in future studies were included in this analysis. The outcome of prevalent malaria infections in the DRC was measured through PCR detection of the *P. falciparum* lactate dehydrogenase gene from DBS samples collected during DHS administration as described previously^12^.

The main exposures were residence within 15 km of a mining concession and residence within 15 km of a logging concession. Additional covariates included individual-level variables for participant age, sex, use of a long-lasting insecticidal net (LLIN), education, and occupation; household variables for wealth, house roofing material, and the ratio of the number of household members using a bed-net to the total number of household members; and cluster variables for elevation, temperature, precipitation, vegetation, percentage of land cover identified as cropland, grassland, forest, and flooded/swamp land. All individual and household variables were obtained through the DHS. Occupation was recoded such that the manual labor and army category included laborers in mining and logging industries. Cluster variables were extracted from various satellite imagery platforms and other spatial datasets; the methods are described in more detail in the “Appendix”. The main exposures were extracted from geographic data sources as described below.

Mining and logging concession data were obtained from the Global Forest Watch online repository^27^. Mining concessions were subset to only include operations that were active or in remediation spanning the DHS study years (2013–2014); logging concessions only included active operations during 2013. Distance to a mining or logging concession was measured from each cluster location to the boundary of a concession. Clusters were considered exposed to mining or logging if they were located within 15 km of a concession. This distance was chosen to account for the estimated 10 km maximum flight distance of a blood-fed mosquito^5^, with an additional 5 km to compensate for boundaries and non-residential land near the concessions. This range also accounts for the 5–10 km random spatial offset implemented by the DHS. Locations of mining and logging concessions along with cluster locations were mapped across the DRC. All maps were created in ArcGIS version 10.7.1, shapefiles for administrative boundaries were obtained from GADM.org.

### Data analysis

Characteristics of the study population were evaluated across quantiles of *P. falciparum* cluster prevalence and grouped by individual, household, and cluster level variables. To further examine distributions of malaria interventions and risk factors such as age, sex, LLIN use, occupation, household wealth, and household roof materials by mining and logging exposure, we compared mining exposed and logging exposed clusters with mining and logging doubly unexposed clusters stratified by urban and rural residence.

We then modelled the prevalence odds of malaria across the DRC using hierarchical logistic regression models to account for the nested structure of the DHS data and to allow for inclusion of spatially varying effects. Models were implemented in a Bayesian framework using Integrated Nested Laplace Approximation (INLA) and stochastic partial differential equations for spatial effects^28^. In all models, we included two separate indicator terms for proximity to a mining concession and to a logging concession; since these areas are non-overlapping, the referent condition for each of these exposures is therefore locations exposed neither to mining nor to logging.

The model fitting process followed two approaches. The first approach evaluated population-level effects of mining and logging on malaria prevalence adjusting for covariates and accounting for cluster-level random effects, which were assumed to vary independently across clusters. The second approach retained covariates and the cluster-level random intercept from the first model and additionally incorporated a spatial field to account for confounding due to space. For the spatial approach, two models were constructed. The first included a spatially varying intercept which borrowed information from neighboring cluster locations assuming a Gaussian random field. The second spatial model explored possible residual confounding due to environmental covariates by allowing spatially varying slopes for temperature, precipitation, vegetation, elevation, and land cover classes while including both independently and spatially varying intercepts across clusters. We introduced spatially varying slopes to account for the unobserved vector population across the DRC. Temperature, precipitation, vegetation, elevation, and various land cover classes have been shown to influence vector composition, survival, and competence for *P. falciparum*^5,23,25^, and associations with these covariates may vary due to their effects on the unobserved vector population. Using the spatial modelling approach, we also constructed a smoothed predicted prevalence map of malaria across the DRC, additional details are in the “Appendix”.

For all models, confounding variables were selected based on a directed acyclic graph analysis and retained for adjustment if the 95% uncertainty interval (UI) of the variable excluded the null. Variables were coded as they were presented in the DHS with the exception of collapsing wealth into moderate or higher versus low wealth and recategorization of occupation as: professional, sales, or services; not working; manual labor or army; and agricultural work. All environmental variables were coded as continuous and scaled. Land cover variables were coded in intervals of 10 percentage points. Model comparison was done using Deviance Information Criterion (DIC), with the best fitting model having the smallest DIC^29^. All models were run using the ‘INLA’ package in R version 4.0.4^28^, additional details are described in the “Appendix”.

Differences in urban and rural residence were considered an important potential source of bias. Urban residence has been associated with lower prevalence of malaria due to many factors including different vector habitats, better access to healthcare, improved housing construction, and overall higher wealth^4,12^. To address possible bias introduced by urban residence, we stratified all models by urban and rural residence based on the DHS classification of clusters as urban or rural.

A discrete set of confounding variables was identified from fixed effect models for mining and logging in rural and urban areas. The final adjustment set included age, sex, LLIN use, household wealth, temperature, precipitation, vegetation, and elevation. These variables had statistical or substantive significance and were adjusted for in all consecutive analyses.

Ethical approval for this study was obtained from the University of North Carolina Institutional Review Board (UNC IRB# 20-3175) and the Kinshasa School of Public Health. Informed consent was obtained from all participants and all methods were conducted in accordance with guidelines and regulations set forth by the UNC IRB and the Kinshasa School of Public Health.

## Results

**Figure 1:**
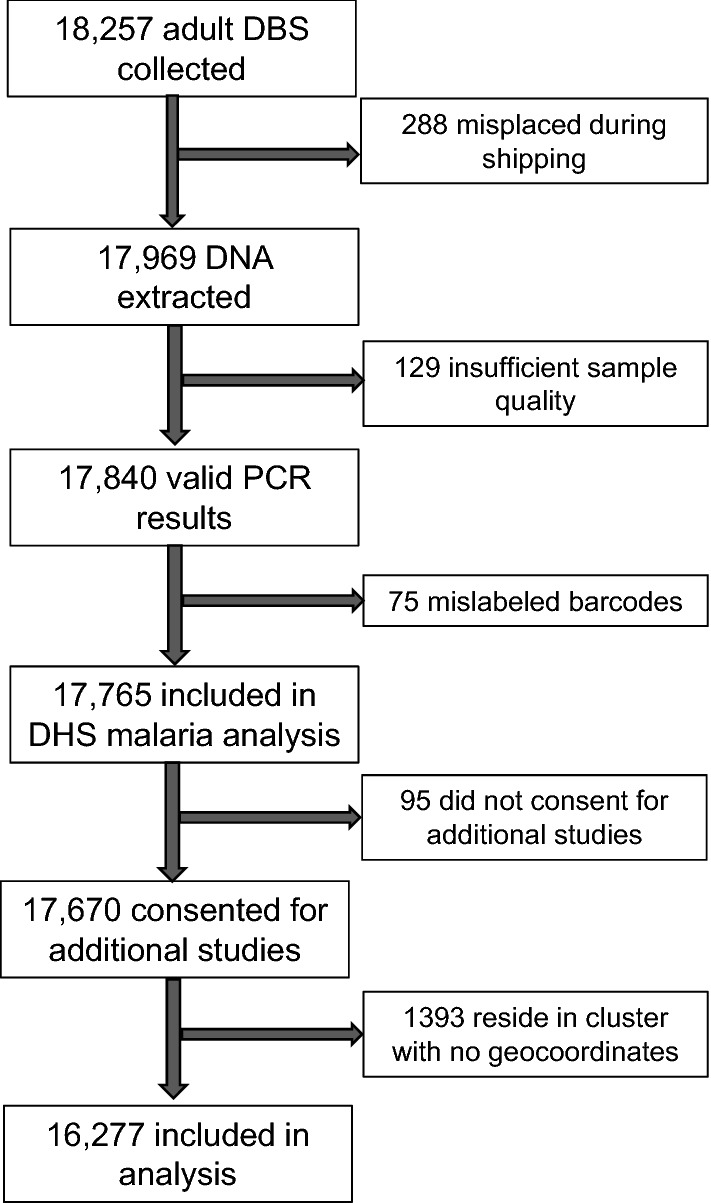
Selection of adult participants in the 2013–2014 DRC DHS into the final analysis.

**Figure 2:**
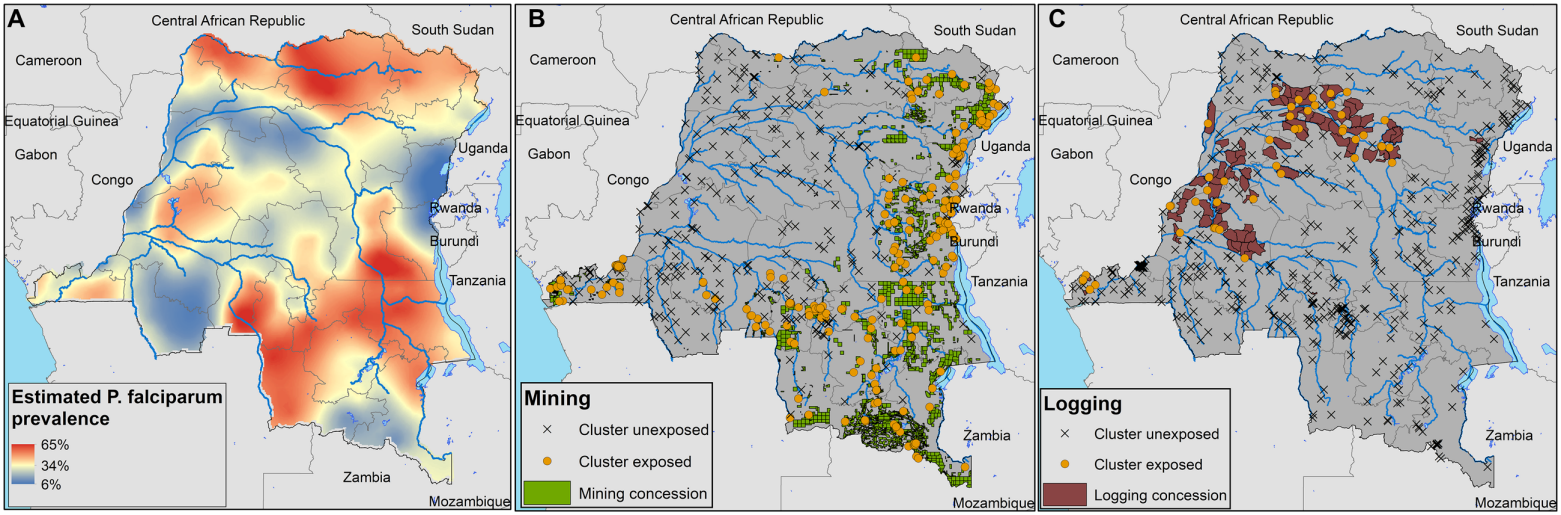
Estimated prevalence of *P. falciparum* across the DRC (**A**), and locations of mining concessions (**B**) and logging concessions (**C**) with clusters exposed to each industry marked by a yellow circle and unexposed clusters marked by a black ‘x’. Maps were generated in ArcGIS version 10.7.1.

A total of 16,277 adults across 489 clusters had available *P. falciparum* PCR results, consented for participation in downstream analyses, and had available cluster location data (Fig. 1). The prevalence of malaria in rural areas was 36.0% (95% UI 35.0, 36.9%) and in urban areas was 28.5% (95% UI 27.4, 29.6%). A predicted surface map of malaria prevalence across the DRC indicated higher prevalence in the north and south-eastern regions and lower prevalence along the eastern border and throughout the Congo River basin (Fig. 2a). The median age of participants was 28 years (IQR: 20–38) and did not differ significantly across quantiles of *P. falciparum* cluster prevalence (Table 1). Lower quantiles of prevalence correlated with higher individual LLIN use, higher education, more skilled work (professional, sales, and services) and less agricultural work. Households with a higher wealth index, finished roof material, and a higher proportion of household nets per person were more likely to fall into a lower quantile of *P. falciparum* prevalence. Lower cluster level precipitation, vegetation, and forest coverage and higher population density were also associated with lower cluster *P. falciparum* prevalence.

**Table 1:** Characteristics of the study population stratified by quartile of *P. falciparum* cluster prevalence and grouped by level of observation (individual, household, and cluster).

	*P. falciparum* PCR prevalence				
	Overall	0–13%	14–30%	31–50%	51–88%
**Individual level**					
n	16,277	4060	3968	3985	4264
Age	28 [20, 38]	28 [20, 37]	28 [20, 38]	28 [20, 38]	28 [20, 38]
Male (%)	7788 (48)	1890 (47)	1940 (49)	1884 (47)	2074 (49)
LLIN use (%)	8449 (52)	2264 (56)	2008 (51)	2100 (53)	2077 (49)
Education (%)					
None	1824 (11)	452 (11)	344 (9)	460 (12)	568 (13)
Primary	5273 (32)	1085 (27)	1188 (30)	1299 (33)	1701 (40)
Secondary and higher	9167 (56)	2520 (62)	2431 (61)	2222 (56)	1994 (47)
Missing	13 (0)	3 (0)	5 (0)	4 (0)	1 (0)
Occupation (%)					
Professional/sales/services	3231 (20)	943 (23)	875 (22)	700 (18)	713 (17)
Not working	3513 (22)	1070 (26)	887 (22)	821 (21)	735 (17)
Manual labor or army	1642 (10)	480 (12)	432 (11)	387 (10)	343 (8)
Agricultural work	7353 (45)	1445 (36)	1662 (42)	1930 (48)	2316 (54)
Missing	538 (3)	122 (3)	112 (3)	147 (4)	157 (4)
**Household level**					
n	7264	1788	1748	1769	1959
Wealth (moderate or higher) (%)	3882 (53)	1124 (63)	976 (56)	895 (51)	887 (45)
Finished roof (%)	2329 (32)	863 (48)	625 (36)	429 (24)	412 (21)
Household net ratio	0.2 [0.0, 0.4]	0.3 [0.1, 0.4]	0.2 [0.0, 0.4]	0.2 [0.0, 0.4]	0.2 [0.0, 0.4]
1 net per 2 people (%)	1493 (21)	421 (24)	343 (20)	378 (21)	351 (18)
**Cluster level**					
n	489	120	118	118	133
Rural (%)	332 (68)	71 (59)	76 (64)	87 (74)	98 (74)
Mining within 15 km (%)	234 (48)	64 (53)	55 (47)	56 (47)	59 (44)
Distance to mining (km)	17 [3, 80]	11 [4, 94]	18 [4, 102]	18 [5, 91]	19 [2, 51]
Logging within 15 km (%)	48 (10)	12 (10)	12 (10)	13 (11)	11 (8)
Distance to logging (km)	182 [59, 290]	186 [58, 266]	142 [57, 265]	179 [59, 280]	185 [60, 326]
Elevation (m)	584 [401, 855]	679 [389, 1473]	519 [386, 747]	592 [422, 804]	601 [422, 759]
Temperature (degrees above 16 °C)	12 [11, 14]	12 [9, 13]	12 [11, 14]	12 [11, 14]	13 [11,14]
Precipitation (mm)	5 [4, 6]	4 [3, 6]	5 [3, 6]	5 [3, 6]	6 [4, 7]
Vegetation (EVI)	4555 [3953, 5084]	4274 [2882, 4830]	4484 [3697, 5037]	4652 [4134, 5171]	4756 [4278, 5165]
Percent cropland coverage	11 [3, 32]	12 [4, 39]	12 [3, 30]	11 [3, 27]	9 [2, 33]
Percent grassland coverage	6 [2, 22]	6 [1, 21]	5 [2, 17]	7 [1, 21]	8 [4, 24]
Percent forest coverage	46 [13, 74]	20 [6, 56]	37 [11, 79]	55 [24, 80]	53 [30, 71]
Percent flooded/swamp coverage	4 [0, 15]	4 [0, 19]	4 [0, 11]	2 [0, 14]	4 [0, 14]

Data are n (%) or median [IQR].

*PCR* polymerase chain reaction, *IQR* interquartile range, *LLIN* long-lasting insecticidal net, *EVI* enhanced vegetation index.

Comparisons of malaria risk factors by mining and logging exposure revealed that LLIN use was lowest among individuals residing in mining exposed clusters and highest among individuals in logging exposed clusters (Table 2). Wealth was considerably higher among mining exposed households and in urban areas with 93% of mining exposed urban households reporting moderate or higher wealth. Household wealth was lowest in clusters not exposed to mining or logging in rural areas, but in urban areas, household wealth was lower among those exposed to logging than among doubly unexposed households. This is consistent with findings that mining communities have higher earnings than non-mining communities^9,11^. Finished roofing materials were consistently more common in mining exposed clusters than either logging exposed clusters or doubly unexposed clusters in both rural and urban areas. In general, finished roofs were more common in urban areas, as was higher household wealth.

**Table 2:** Distributions of malaria risk factors for individuals and households residing in mining exposed, logging exposed, and doubly unexposed clusters, stratified by urban/rural status.

	Rural			Urban		
	Mining + logging unexposed	Mining exposed	Logging exposed	Mining + logging unexposed	Mining exposed	Logging exposed
**Individual level**						
n	5272	3956	1108	1583	3938	546
Age	29 [21, 39]	28 [21, 38]	29 [20, 39]	27 [20, 37]	27 [20, 36]	28 [20, 38]
Male (%)	2554 (48)	1878 (47)	559 (50)	779 (49)	1813 (46)	264 (48)
LLIN use (%)	2917 (55)	1982 (50)	598 (54)	880 (56)	1771 (45)	361 (66)
Occupation (%)						
Professional/sales/services	667 (13)	467 (12)	187 (17)	414 (26)	1346 (34)	175 (32)
Not working	896 (17)	606 (15)	174 (16)	458 (29)	1293 (33)	114 (21)
Manual labor or army	280 (5)	268 (7)	34 (3)	231 (15)	767 (19)	78 (14)
Agricultural work	3267 (62)	2504 (63)	679 (61)	416 (26)	383 (10)	160 (29)
Missing	162 (3)	111 (3)	34 (3)	64 (4)	149 (4)	19 (3)
**Household level**						
n	2481	1921	506	644	1538	234
Wealth (moderate or higher) (%)	767 (31)	908 (47)	180 (36)	478 (74)	1432 (93)	162 (69)
Finished roof (%)	188 (8)	429 (22)	57 (11)	282 (44)	1301 (85)	99 (42)

Data are n (%) or median [IQR].

*LLIN* long-lasting insecticidal net.

### Mining and malaria

Mining concessions were prevalent throughout the DRC with 48% of clusters (234/489) located within 15 km of one or more concessions (Fig. 2b). In rural settings, mining was associated with a significant increase in the probability of malaria relative to non-mining and non-logging clusters (POR 1.82, 95% UI 1.35, 2.44) allowing a random intercept for clusters and adjusting for confounding variables. Adding a spatially varying intercept shifted the effect of mining toward the null with a POR of 0.93 (95% UI 0.69, 1.26), adjusting for confounding variables. DIC model fit statistics favored inclusion of the spatially varying intercept (Table 3), suggesting the presence of spatially correlated residual confounding.

**Table 3:** Hierarchical logistic regression model results, non-spatial, and with a spatially varying intercept.

	Random intercept		Spatially varying intercept + random intercept	
	Malaria odds ratio (95% UI)	DIC	Malaria odds ratio (95% UI)	DIC
**Rural**				
Mining	1.82 (1.35, 2.44)		0.93 (0.69, 1.26)	
Logging	0.71 (0.45, 1.12)	11,384.2	0.97 (0.61, 1.53)	11,362.7
**Urban**				
Mining	0.88 (0.57, 1.37)		0.90 (0.54, 1.53)	
Logging	0.58 (0.30, 1.14)	5851.2	0.63 (0.28, 1.44)	5848.0

All models adjusted for age, sex, LLIN use, temperature, precipitation, vegetation, elevation, and household wealth.

*UI* uncertainty interval, *DIC* deviance information criterion.

In urban settings no significant association with mining was detected when accounting for intra-cluster correlation alone (POR: 0.88, 95% UI 0.57, 1.37), or with a spatially varying intercept included (POR: 0.90, 95% UI 0.54, 1.52). Model fit statistics again favored inclusion of the spatially varying intercept over the random cluster intercept alone (Table 3). Addition of spatially varying slopes for environmental variables in both rural and urban areas failed to improve model fit over the spatially varying intercept model (“Appendix”).

### Logging

In the DRC, logging concessions are mostly located in the north-western part of the country, following along the Congo River (Fig. 2c). A total of 48 clusters (9%) were located within 15 km of a logging concession. In rural and urban areas, logging appeared to have a slightly protective association with malaria when controlling for intra-cluster correlation alone; however, the uncertainty intervals for these estimates included the null (Table 3). Inclusion of a spatially varying intercept shifted estimates upward and closer to the null in rural and urban strata with model fit statistics again favoring inclusion of a spatially varying intercept. Adjusting for space, cluster effects, and confounding variables, the prevalence odds ratio of malaria in logging exposed rural areas was 0.97 (0.61, 1.53), and in urban areas was 0.63 (0.28, 1.44).

## Discussion

We evaluated associations between exposure to mining and logging concessions and malaria prevalence among Congolese adults. Using population representative malaria survey results and geographic data for mining and logging concessions, our results revealed complex relationships between mining, logging, malaria, and space. Without accounting for space, we found malaria prevalence was higher among individuals in rural areas exposed to mining concessions than among individuals not exposed to mining or logging. When accounting for spatial relationships, exposure to mining and logging operations was unassociated with malaria prevalence. The difference in effect estimates for mining and malaria between the nonspatial and spatial modelling approaches suggests two important conclusions. First, malaria prevalence is high among many individuals in rural areas exposed to mining concessions, a pattern that is also apparent in the predicted malaria prevalence map in Fig. 2a. Areas of elevated prevalence highlight the importance of targeting malaria control interventions in communities proximal to active mining operations. Secondly, the spatial models suggest that the high prevalence of malaria around mining areas was not explicitly associated with exposure to mining concessions themselves. This suggests that mining concessions tend to be located in areas of high malaria prevalence due to factors not measured in these analyses.

The mining sector in the DRC is large and has a complex history of human migration, private and governmental interests, and rapid expansion. In the context of malaria, these features may have led to a mixing of parasite populations^30^ and different approaches to malaria prevention interventions and vector control^9,13^—all of which could have contributed to the spatial confounding of mining and malaria that we observed. Spatial variation in parasite genotypes, particularly related to antimalarial resistance, has been shown throughout the DRC, with clusters of drug resistant strains persisting in and around the eastern border and south-central mining regions^30^. The presence of these genotypes further complicate malaria control and prevention efforts in this high transmission area. Additionally, high rates of human in-migration to mining areas contribute to the mixing of parasite populations and can strain local infrastructure, impacting access to healthcare and administration of malaria control interventions^11,13^. We did not collect data on parasite genetics or human migration, and the magnitude of bias that each may have contributed to the effects of mining and logging remains unknown and should be studied further.

Another potential source of spatial confounding is the distribution of malaria vector species. In the DRC, *Anopheles* populations vary widely and have different feeding behaviors, vectorial capacities, and biting rates^5,9^, all of which influence malaria transmission dynamics and can be difficult to accurately model without extensive entomological survey data. We did not have access to data on vector populations, however we used spatially varying terms for environmental and land cover variables commonly associated with vector dynamics to adjust for unobserved vector populations. Inclusion of these terms had little impact on effect estimates for mining and logging, suggesting that these models may have failed to adequately capture the complexity of the relationships between the environment, *Anopheles* vector species, and malaria.

This study has several notable strengths including the use of population representative data for malaria results and geographic data to evaluate mining and logging concessions. To our knowledge, this is the first study to evaluate associations between mining, logging, and malaria using a nationally representative sample of adults in Sub-Saharan Africa. This study also had several limitations. First, the DHS was designed to target households and may have missed individuals engaged in mining or logging labor who reside outside of a traditional household living structure. These individuals are likely at the highest risk of any mining or logging associated malaria and are difficult to capture in malaria surveys. Future studies of mining or logging and malaria focused on individual-level health effects might consider constructing surveys that could capture individuals living outside of traditional household structures. Secondly, the ages of participants in this study ranged from 15 to 59 years, thus it is possible that results could be different for children and the elderly who were not included in our study. Thirdly, the DHS does not collect information on environmental variables, land use, or land cover, therefore these measures were all derived from other data sources and linked to malaria results based on cluster location. Measurement error may be present in the derivation of these additional variables, and may also have been introduced when linking to the DHS clusters and averaging throughout the 10 km cluster buffer. Additionally, our mining and logging datasets relied on government reported data of industrial operations. Smaller scale artisanal mining^7^ and informal timber extraction activities^8^ are prevalent throughout the DRC and may not have been captured by our data sources. Any effect that local mining and forest clearing activities had on malaria would have been misclassified as unexposed and shifted estimates toward the null. Finally, we were unable to evaluate differences by mining practice or types of extracted minerals. Mining concessions in the DRC often extract multiple types of minerals and close geographic clustering of mining concessions engaged in different practices limited our ability to look for differing effects of mining type or minerals on malaria ecologies given the resolution of our data.

Previously published studies of mining and logging on malaria in Africa have mixed results. In the Brong Ahafo region of Ghana, exposure to an area under development for mining was unassociated with malaria among children^15^, similar to our results. In a study of a Zambian copper mine, children living in mining-exposed communities experienced a lower odds of malaria than unexposed children^22^. This finding contrasts with our results and may reflect higher wealth in mining-exposed areas and better healthcare access which may have stronger, more positive impacts on children’s health than adults. Other studies of mining and malaria in sub-Saharan Africa have focused on malaria control interventions implemented within communities around mining concessions^13,21^. While these studies do not compare malaria infections between communities that are exposed and unexposed to mining, they illustrate successful engagement of the mining sector in malaria control and prevention^13,14,21^, efforts which should continue to be supported. In the DRC, large mining concessions have historically provided insecticide treated nets and administered indoor residual spraying to reduce malaria transmission^11,13^. In Fungurume, a town in southern DRC with active copper and cobalt mining operations, entomological surveillance conducted contemporaneously with the DHS found that the local mosquito population had collapsed, likely because of vector control^5^. We assessed for differences in the relationship between malaria and mining across individual mining concessions in this study, however we did not detect any significant variation. Finally, the results of our logging analysis align with a recent ecological study of deforestation and malaria in sub-Saharan Africa, which found malaria prevalence unassociated with increasing levels of deforestation^24^. While our results are similar to other null findings in Africa, they contrast with strong relationships between mining and malaria^16,17^ and deforestation and malaria^10,19^ in South America, suggesting that the effects of resource extraction on malaria may depend on regional malaria ecologies. Because of this, our results may have limited generalizability outside of the DRC and sub-Saharan Africa.

In our evaluation of mining, logging, and malaria in the DRC we found that mining and logging concessions did not have a detectable effect on malaria prevalence when controlling for spatial confounding. While these results suggest that the mining and logging industries alone may not intensify the burden of malaria in the DRC, it is important to note that malaria prevalence remains high across the country and is further elevated in many areas with active mining operations. Therefore, transmission of malaria should continue to be monitored throughout the DRC as a whole, with particular attention to areas with larger scale resource extraction which should remain a high priority for malaria control interventions especially as demand for natural resources grows and these activities expand.

## Supplementary Information

Supplementary Information.

## Data Availability

The datasets generated and analyzed during the present study are available from the corresponding author upon request.
