# Supplementary material for: Impact of extractive industries on malaria prevalence in the Democratic Republic of the Congo: a population-based cross-sectional study
Source: Sci Rep. 2022 Feb 2;12:1737. doi: 10.1038/s41598-022-05777-9 (PMC8810856; doi:10.1038/s41598-022-05777-9)
Supplement: Supplementary file 1 — Supplementary Information. [file 41598_2022_5777_MOESM1_ESM.docx]

**Supplementary appendix:** Impact of extractive industries on malaria prevalence in the Democratic Republic of the Congo: a population-based cross-sectional study

Cedar L. Mitchell, Mark M. Janko, Melchior K. Mwandagalirwa, Antoinette K. Tshefu, Jessie K. Edwards, Brian W. Pence, Jonathan J. Juliano, Michael Emch

Contents

[**S1. STROBE Statement for cross-sectional studies** 26](#_Toc74143105)

[**S2. Environmental and land cover data extraction** 31](#_Toc74143106)

[**S3. Model structure and development** 32](#_Toc74143107)

[**S3.1 Bayesian deterministic model** 32](#_Toc74143108)

[**S3.2 Bayesian predictive model and estimated prevalence map** 35](#_Toc74143109)

[**S4. Sensitivity analyses** 36](#_Toc74143110)

[**S4.1 Spatial slopes** 36](#_Toc74143111)

[**S4.2 Temporal lags for temperature, precipitation, and vegetation** 39](#_Toc74143112)

[**S5. References:** 41](#_Toc74143113)

# **S1. STROBE Statement for cross-sectional studies**

|  | Item No | Recommendation |
| --- | --- | --- |
| **Title and abstract** | 1 | (*a*) Indicate the study’s design with a commonly used term in the title or the abstract  **The title states that this is a cross-sectional study** |
|  |  | (*b*) Provide in the abstract an informative and balanced summary of what was done and what was found  **The abstract includes a summary of rationale for the study, descriptions of the data sources used, statistical methods, results, and relevance of the findings.** |
| Introduction | | |
| Background/rationale | 2 | Explain the scientific background and rationale for the investigation being reported  **We describe the importance of malaria as a public health burden in Africa, the importance of mining and logging as extractive industries in the region, and their propensity for disrupting local environments as they relate to malaria transmission. We highlight that few studies have been conducted to evaluate the role of mining or logging in malaria transmission in Africa, rather much of the literature stems from studies in South America and may not be translatable to Africa.** |
| Objectives | 3 | State specific objectives, including any prespecified hypotheses  **We state that the study was conducted to evaluate for associations between mining and logging concessions and malaria prevalence in the Democratic Republic of the Congo.** |
| Methods | | |
| Study design | 4 | Present key elements of study design early in the paper  **The study design is described in the first section of the methods detailing use of the 2013 DHS as the primary data source and linkage with geographic data sources for mining and logging exposure and extraction of environmental covariates.** |
| Setting | 5 | Describe the setting, locations, and relevant dates, including periods of recruitment, exposure, follow-up, and data collection  **We describe that we used population representative data for the DRC from the DHS for the study during 2013- 2014. We also report that mining and logging data were extracted only for the year that the DHS was initially administered to ensure temporality and describe how environmental variables were summarized around the month of the DHS interviews.** |
| Participants | 6 | (*a*) Give the eligibility criteria, and the sources and methods of selection of participants  **We provide detail on how participants were selected for the DHS including selection of participants into our analysis. Exclusion of participants from the initial DHS for our study is outlined in detail in Figure 1.** |
| Variables | 7 | Clearly define all outcomes, exposures, predictors, potential confounders, and effect modifiers. Give diagnostic criteria, if applicable  **We describe how the main exposures were derived from mining and logging geographic datasets and clearly state which demographic and environmental variables were also included as covariates. The outcome is clearly specified and a brief description of the diagnosis of *P. falciparum* is included with a reference to a paper that describes the diagnostic methods in more detail.** |
| Data sources/ measurement | 8* | For each variable of interest, give sources of data and details of methods of assessment (measurement). Describe comparability of assessment methods if there is more than one group  **We state whether variables were derived from the DHS or geographic data. For variables derived from geographic data, we provided the specific data source and detail regarding how each variable was measured and summarized, when applicable.** |
| Bias | 9 | Describe any efforts to address potential sources of bias  **We describe use of a directed acyclic graph to identify potential sources of confounding bias. We also assess for possible measurement error due to temporal lags in effect for several environmental variables through a sensitivity analysis as described in the Appendix. We also evaluate for bias due to spatial autocorrelation in the data and discuss the implications of spatial confounding in detail in the discussion.** |
| Study size | 10 | Explain how the study size was arrived at  **We included a diagram illustrating the number of participants excluded from the study and reasons for exclusion to arrive at the final sample size in Figure 1.** |
| Quantitative variables | 11 | Explain how quantitative variables were handled in the analyses. If applicable, describe which groupings were chosen and why  **We state that variables derived from the DHS were coded as they were presented in the DHS and detail and exceptions. We also mentioned that all environmental variables were coded as continuous and scaled to reduce collinearity.** |
| Statistical methods | 12 | (*a*) Describe all statistical methods, including those used to control for confounding  **We describe the statistical approaches and models that were used with additional detail in the Appendix. We mention that variables were included in regression models to control for confounding and that models were stratified by urban/rural residence to reduce bias due to residual urban/rural effects.** |
|  |  | (*b*) Describe any methods used to examine subgroups and interactions  **We state that models were stratified by urban/rural residence and describe how variables were evaluated for spatial interactions in the Appendix.** |
|  |  | (*c*) Explain how missing data were addressed  **We describe participants excluded for missing outcome or location data in the participant selection chart in Figure 1.** |
|  |  | (*d*) If applicable, describe analytical methods taking account of sampling strategy  **We describe the use of a random intercept to correct for intra-cluster correlation introduced by the DHS sampling design.** |
|  |  | (*e*) Describe any sensitivity analyses  **In the Appendix we summarize a sensitivity analysis for temporal bias in the environmental measures of temperature, precipitation, and elevation. We also describe a sensitivity analysis of spatially varying slopes for environmental and land cover covariates.** |
| Results | | |
| Participants | 13* | (a) Report numbers of individuals at each stage of study—eg numbers potentially eligible, examined for eligibility, confirmed eligible, included in the study, completing follow-up, and analysed  **We describe the selection of participants into the study and detail numbers of individuals excluded for each reason in the participant selection chart in Figure 1.** |
|  |  | (b) Give reasons for non-participation at each stage  **We do not have information regarding why participants may not have participated in the DHS, however we do provide reasons for exclusion of participants due to sample or location data availability.** |
|  |  | (c) Consider use of a flow diagram  **We include a flow diagram for participant selection in Figure 1.** |
| Descriptive data | 14* | (a) Give characteristics of study participants (eg demographic, clinical, social) and information on exposures and potential confounders  **We provide demographic, behavioural, and environmental characteristics of participants and their locations in Table 1 and summarize findings at the start of the results section. We also describe characteristic of participants by exposure and urban/rural status for several potential confounders in Table 2.** |
|  |  | (b) Indicate number of participants with missing data for each variable of interest  **For any variables with missing data, we specify the number of participants with missing data in Table 1.** |
| Outcome data | 15* | Report numbers of outcome events or summary measures  **We report the prevalence of *P. falciparum* malaria in urban and rural areas and report numbers of participants in each quantile of *P. falciparum* cluster prevalence and for each variable in Table 1.** |
| Main results | 16 | (*a*) Give unadjusted estimates and, if applicable, confounder-adjusted estimates and their precision (eg, 95% confidence interval). Make clear which confounders were adjusted for and why they were included  **We provide estimates that are adjusted for by fixed effect confounders and estimates that confounder-adjusted using fixed effects and spatially varying effects. We specify why confounders were adjusted for and why spatial effects were also adjusted for. All estimates include 95% Bayesian uncertainty intervals.** |
|  |  | (*b*) Report category boundaries when continuous variables were categorized  **Continuous variables were coded as continuous and scaled, we did not categorize any continuous variables in this study.** |
|  |  | (*c*) If relevant, consider translating estimates of relative risk into absolute risk for a meaningful time period  **It is not relevant to translate relative risk estimates to the additive scale in our study.** |
| Other analyses | 17 | Report other analyses done—eg analyses of subgroups and interactions, and sensitivity analyses  **We report sensitivity analyses in the Appendix.** |
| Discussion | | |
| Key results | 18 | Summarise key results with reference to study objectives  **We restate the study objectives in the discussion and promptly present the main results of the study.** |
| Limitations | 19 | Discuss limitations of the study, taking into account sources of potential bias or imprecision. Discuss both direction and magnitude of any potential bias  **We discuss limitations of the study and postulate possible sources of residual confounding bias, weaknesses in the data sources, and sources of possible measurement error.** |
| Interpretation | 20 | Give a cautious overall interpretation of results considering objectives, limitations, multiplicity of analyses, results from similar studies, and other relevant evidence  **We interpret the results in the context of the study and are careful to not overstate implications of our findings. We reflect on limitations of our analysis and compare our results with those of similar studies.** |
| Generalisability | 21 | Discuss the generalisability (external validity) of the study results  **We state that our results may have limited generalizability outside of the study area due to regional differences in malaria ecologies.** |
| Other information | | |
| Funding | 22 | Give the source of funding and the role of the funders for the present study and, if applicable, for the original study on which the present article is based  **We present the source of funding and disclaim that the funders had no role in the present study.** |

# **S2. Environmental and land cover data extraction**

Measures of land surface temperature and enhanced vegetation index (EVI) were obtained from the MODIS satellite platform. Precipitation data were extracted from the CHIRPS dataset using the R package ‘chirps’. Values for temperature, vegetation (EVI), and precipitation were averaged monthly. A time delay of one month preceding the month of interview was used for temperature and vegetation, precipitation values had no time lag. A sensitivity analysis was conducted to evaluate the sensitivity of the results to different time lags and is summarized in section S4.2 of the Appendix. Elevation was derived from the SRTM dataset through the ‘raster’ package in R. Landcover data were obtained through the ESA Climate Change Initiative land cover dataset for 2013 with land cover classes aggregated into 4 land cover types: cropland, grassland/shrubland, forest, and flooded/swamp. All environmental variables were extracted within a 10km buffer around the DHS cluster locations and averaged to account for the random cluster location offset.

# **S3. Model structure and development**

## **S3.1 Bayesian hierarchical model**

We used hierarchical logistic regression models to estimate the prevalence odds of malaria in the DRC for 2013-2014. Our outcome of interest, malaria prevalence odds, was a binary event and we used a hierarchical Bayesian model for estimation of individual level outcomes adjusting for predictors at the individual and cluster levels. We evaluated three distinct possible data structures: 1) the presence of intra-cluster correlation among survey clusters, 2) the presence of general spatial confounding, and 3) spatial variability in predictor-response relationships. To address the first data structure, we incorporated a random intercept for the DHS survey clusters as denoted below:

$logit\left( p_{ij} \right)= \boldsymbol{x}_{ij}^{T}\boldsymbol{\beta+}u_{j}$

$$\boldsymbol{U}\sim N(\boldsymbol{0},\sigma_{u}^{2}\boldsymbol{I})$$

Here, we estimated the log-odds of malaria prevalence ${(p}_{ij})$ for individual *i* in cluster *j* using$\boldsymbol{x}_{ij}$ to represent a vector of covariates at the individual and cluster levels, and $u_{j}$ as the cluster- level varying intercept.

The second and third data structures required approximation of a continuous spatial field. Using the stochastic partial differential equation based approach from Lindgren and Rue,^1^ we fit a continuous process across space from a mesh of discrete spatial points informed by the locations of the DHS survey clusters stratified by rural and urban areas (Supplemental Figure 1). We assessed for spatial confounding using a spatially varying intercept as described below:

$logit\left( p_{ij} \right)= x_{ij}\beta\boldsymbol{+}u_{j}+s_{j}$

$$\boldsymbol{U}\sim N(\boldsymbol{0},\sigma_{u}^{2}\boldsymbol{I})$$

$$\boldsymbol{S}\sim N(\boldsymbol{0},\sigma_{s}^{2}\boldsymbol{\Sigma}\left( \phi\right))$$

The spatially varying intercept, ***S***, was defined by a set of priors with a spatial range of 1/5 the size of the spatial field with a 50% probability of a shorter realized range, and a variance of 0.5 with a 50% probability of exceeding the prior variance. A random intercept for survey clusters, ***U***, was also included to control for residual within cluster correlation structures. We tested inclusion of the spatially varying intercept alone and the model fit statistics overwhelmingly supported inclusion of both the spatially varying intercept and the cluster random intercept (data not shown).


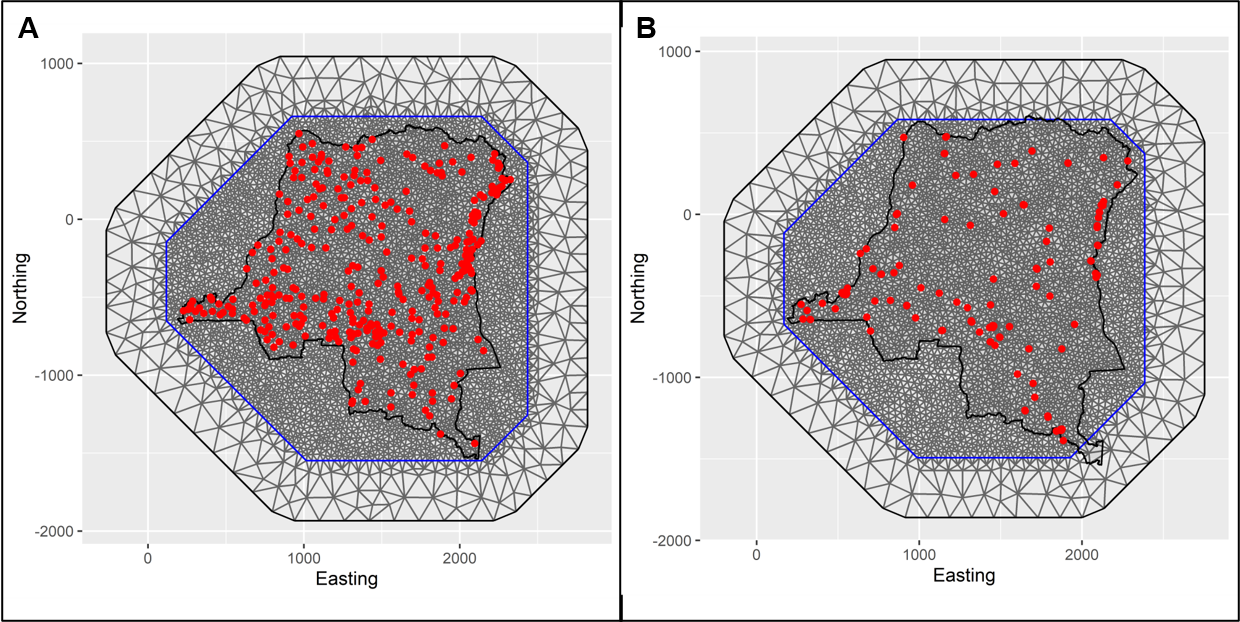


**Supplemental figure 1.** Spatial mesh formations for rural (A) and urban (B) clusters in the DRC DHS. Cluster locations are marked by red points.

In the final assessment, we incorporated spatially varying slopes to flexibly model variations in predictor-response relationships across space.

$$logit\left( p_{ij} \right)= \boldsymbol{x}_{ij}^{T}\boldsymbol{\beta+}u_{j}+s_{j}+{z_{j}\eta}_{j}$$

$$\boldsymbol{U}\sim N(\boldsymbol{0},\sigma_{u}^{2}\boldsymbol{I})$$

$$\boldsymbol{S}\sim N(\boldsymbol{0},\sigma_{s}^{2}\boldsymbol{\Sigma}\left( \phi\right))$$

$$\boldsymbol{\eta}\sim N(\boldsymbol{0},\sigma_{\eta}^{2}\boldsymbol{\Sigma}\left( \phi_{\eta} \right))$$

Here, *z_j_* is the cluster-level covariate of interest and $\eta_{j}$ is the spatially varying random effect for all individuals in the specified cluster. The cluster-level varying intercept and spatial intercept remain specified as before to control for intra-cluster correlation and spatial confounding.

## **S3.2 Bayesian predictive model and estimated prevalence map**

To estimate the prevalence of *P. falciparum* across the study site, we used R-INLA to predict a spatial surface using prevalence estimates from our data. We constructed a prediction grid of 450x450 locations within the borders of the DRC and linked the prediction locations to a spatial mesh constructed from the DHS cluster locations for the full study. Malaria prevalence predictions were jointly calculated with prevalence estimates drawn from our primary dataset. Posterior means for predicted malaria prevalence were extracted to the prediction nodes and plotted in R using the ‘Lattice’ package^2^ and exported to ArcMap version 10.7.1 for mapping. Supplemental Figure 2 illustrates the predicted prevalence means and standard deviation values across the study site.


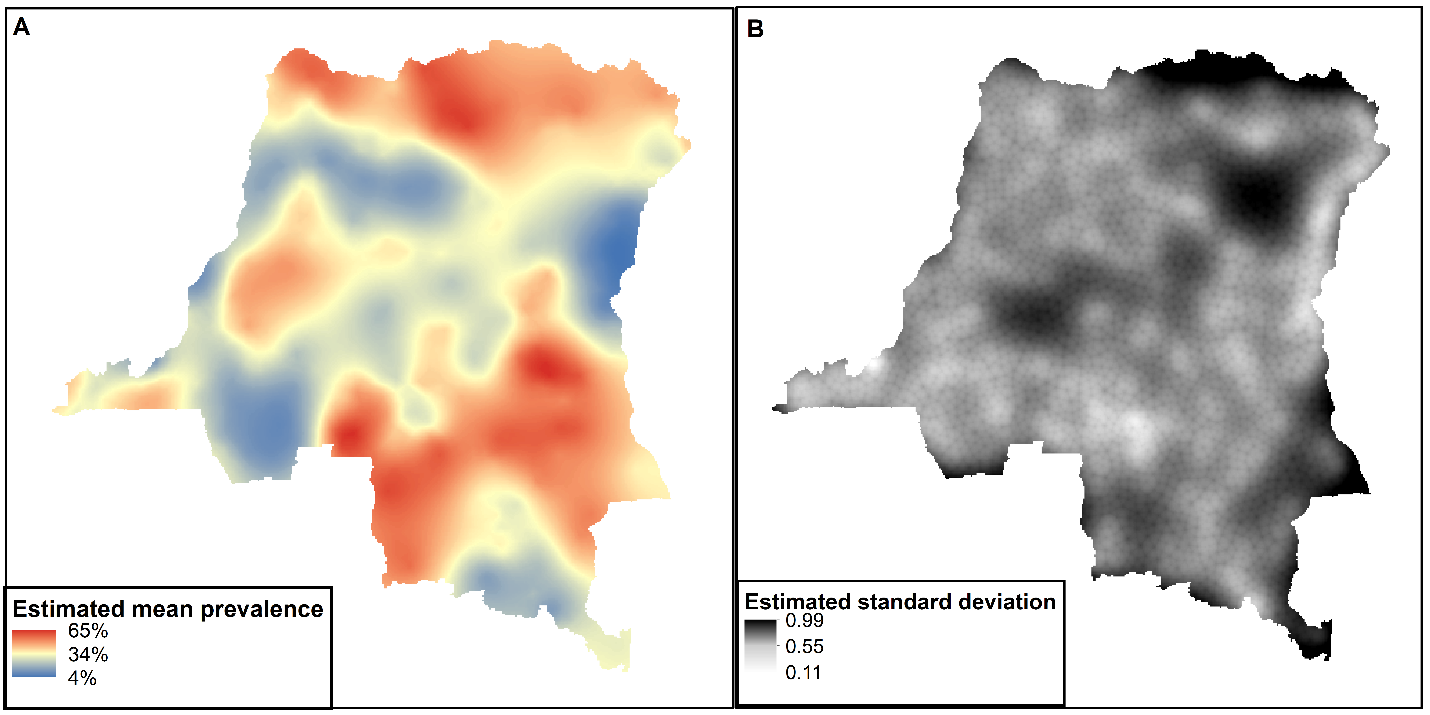


**Supplemental figure 2.** Predictive map of *P. falciparum* prevalence means across the DRC (Panel A) and predicted standard deviations (Panel B).

# **S4. Sensitivity analyses**

## **S4.1 Spatial slopes**

To assess the potential for spatially varying confounders, we tested a spatial slope term for each environmental and land cover predictor individually. Relationships between various environmental and land cover variables and malaria likely vary across space.^3,4^ We flexibly modelled spatial effects of temperature, precipitation, vegetation, elevation, forest cover, grassland, cropland, and flooded/swamp lands on malaria prevalence in rural and urban areas while controlling for random cluster effects and fixed effects for mining, logging, age, sex, LLIN use, household wealth, temperature, precipitation, vegetation, and elevation. None of the spatially varying environmental (Supplemental table 1) or land cover (Supplemental table 2) variable terms significantly shifted effect estimates for mining or logging in rural and urban settings. Model fit statistics slightly favored incorporation of a random slope for temperature in rural settings and inclusion of a random slope for flooded/swamp lands in urban areas. However, in both instances, model fit was improved by a marginal number of points.

**Supplemental table 1.** Hierarchical logistic regression model results including spatially varying slopes for environmental predictors.

|  | **Temperature^a^** | | **Precipitation^b^** | | **Vegetation^c^** | | **Elevation^d^** | |
| --- | --- | --- | --- | --- | --- | --- | --- | --- |
|  | Malaria odds ratio (95% UI) | DIC | Malaria odds ratio (95% UI) | DIC | Malaria odds ratio (95% UI) | DIC | Malaria odds ratio (95% UI) | DIC |
| **Rural** |  |  |  |  |  |  |  |  |
| Mining | 0.92 (0.68, 1.25) |  | 0.92 (0.68, 1.26) |  | 0.92 (0.68, 1.25) |  | 0.91 (0.67, 1.24) |  |
| Logging | 0.95 (0.60, 1.51) | 11361.7 | 0.94 (0.60, 1.50) | 11363.7 | 0.94 (0.59, 1.48) | 11363.8 | 0.95 (0.60, 1.51) | 11362.4 |
| **Urban** |  |  |  |  |  |  |  |  |
| Mining | 0.92 (0.54, 1.56) |  | 0.91 (0.54, 1.55) |  | 0.87 (0.52, 1.48) |  | 0.87 (0.52, 1.47) |  |
| Logging | 0.63 (0.27, 1.46) | 5847.3 | 0.64 (0.28, 1.44) | 5847.3 | 0.62 (0.27, 1.44) | 5848.2 | 0.69 (0.31, 1.53) | 5847.4 |

^a^ Adjusted for fixed effects of age, sex, LLIN use, precipitation, vegetation, elevation, and household wealth.

^b^ Adjusted for fixed effects of age, sex, LLIN use, temperature, vegetation, elevation, and household wealth.

^c^ Adjusted for fixed effects of age, sex, LLIN use, temperature, precipitation, elevation, and household wealth.

^d^ Adjusted for fixed effects of age, sex, LLIN use, temperature, precipitation, vegetation, and household wealth.

**Supplemental table 2.** Hierarchical logistic regression model results including spatially varying slopes for land cover classes.

|  | **Forest** | | **Grassland** | | **Cropland** | | **Flooded** | |
| --- | --- | --- | --- | --- | --- | --- | --- | --- |
|  | Malaria odds ratio (95% UI) | DIC | Malaria odds ratio (95% UI) | DIC | Malaria odds ratio (95% UI) | DIC | Malaria odds ratio (95% UI) | DIC |
| **Rural** |  |  |  |  |  |  |  |  |
| Mining | 0.92 (0.68, 1.26) |  | 0.93 (0.69. 1.27) |  | 0.92 (0.67, 1.25) |  | 0.90 (0.67, 1.22) |  |
| Logging | 0.94 (0.59, 1.50) | 11362.5 | 0.98 (0.62, 1.56) | 11363.1 | 0.99 (0.62, 1.58) | 11362.5 | 0.92 (0.58, 1.47) | 11362.1 |
| **Urban** |  |  |  |  |  |  |  |  |
| Mining | 0.84 (0.51, 1.37) |  | 0.92 (0.54, 1.56) |  | 0.90 (0.53, 1.53) |  | 0.89 (0.53, 1.48) |  |
| Logging | 0.70 (0.32, 1.55) | 5846.8 | 0.64 (0.28, 1.46) | 5848.1 | 0.56 (0.23, 1.31) | 5847.5 | 0.61 (0.25, 1.48) | 5846.5 |

All models adjusted for fixed effects of age, sex, LLIN use, temperature, precipitation, vegetation, elevation, and household wealth.

## **S4.2 Temporal lags for temperature, precipitation, and vegetation**

Temperature, precipitation, and vegetation have all been shown to relate to malaria prevalence following various durations of time.^3-5^ Typically, values for each variable are measured over a monthly period and are evaluated for the month of the survey or lagged by 1-month intervals up to 3 months prior to survey administration. We chose to average the values for each environmental predictor spanning the month of interview, and averaged during the first month prior to the survey month, the second month prior, and the third month prior. Decisions for which time lag to use, if any, were based on substantive knowledge (i.e. how long does precipitated water remain available for vector breeding and survival needs), as well as effect size and precision, favoring estimates that were further from the null and with more narrow uncertainty intervals.

To evaluate the effect that differing temporal delays might have on the results of our study, we evaluated the full range of temporal delays (0-3 months prior to the month of interview) for temperature, precipitation, and vegetation measures. The results are shown in Supplemental Figure 3 comparing the effect estimates for mining and logging from our final model (referent) with the full range of time lags for temperature, precipitation, and vegetation. In rural and urban areas, we found that different temporal delays introduced little variation in the effect estimates for mining and logging and had no significant effects.


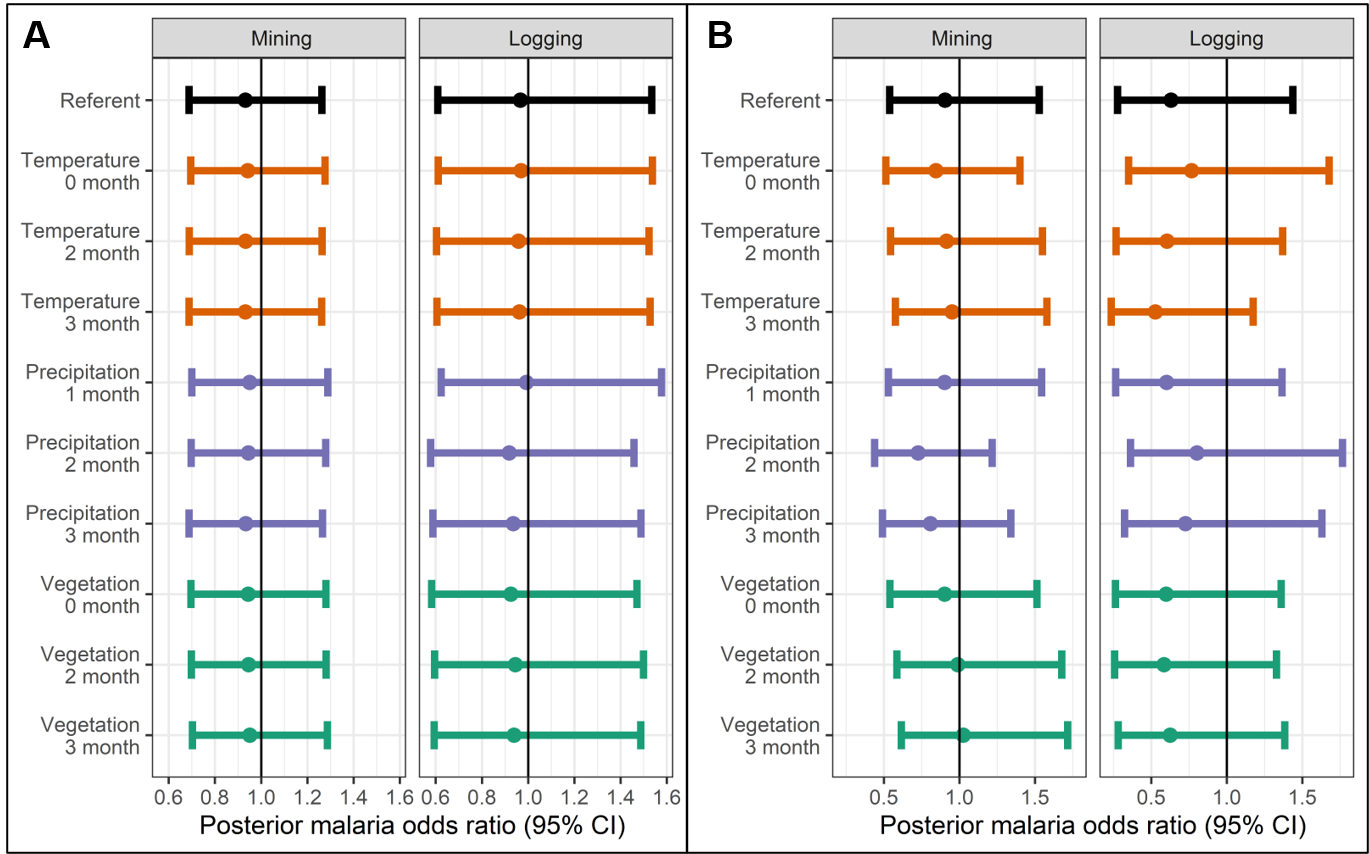


**Supplemental Figure 3.** Effect estimates for mining and logging comparing our final model (referent) with models incorporating temporal lags of 0-3 months for temperature, precipitation, and vegetation in rural (panel A) and urban (panel B) settings.

# **S5. References:**

1. Lindgren F, Rue H. Bayesian Spatial Modelling with R - INLA. *J Stat Softw* 2015; 63(19). <http://www.jstatsoft.org/v63/i19/>.
2. Sarkar, Deepayan. Lattice: Multivariate Data Visualization with R, Springer. 2008; ISBN: 978-0-387-75968-5. <http://lmdvr.r-forge.r-project.org/>.
3. Weiss DJ, Mappin B, Dalrymple U, Bhatt S, Cameron E, Hay SI, et al. Re-examining environmental correlates of Plasmodium falciparum malaria endemicity: a data-intensive variable selection approach. *Malar J* 2015; 14(1): 68.
4. Janko MM, Irish SR, Reich BJ, Peterson M, Doctor SM, Mwandagalirwa MK, et al. The links between agriculture, Anopheles mosquitoes, and malaria risk in children younger than 5 years in the Democratic Republic of the Congo: a population-based, cross-sectional, spatial study. *Lancet Planet Health.* 2018; 2(2): e74–82.
5. Paaijmans KP, Read AF, Thomas MB. Understanding the link between malaria risk and climate. *Proc Natl Acad Sci USA* 2009; 106(33): 13844–9.
